# Strength Training Prior to Endurance Exercise: Impact on the Neuromuscular System, Endurance Performance and Cardiorespiratory Responses

**DOI:** 10.2478/hukin-2014-0123

**Published:** 2014-12-30

**Authors:** Matheus Conceição, Eduardo Lusa Cadore, Miriam González-Izal, Mikel Izquierdo, Giane Veiga Liedtke, Eurico Nestor Wilhelm, Ronei Silveira Pinto, Fernanda Reistenbach Goltz, Cláudia Dornelles Schneider, Rodrigo Ferrari, Martim Bottaro, Luiz Fernando Martins Kruel

**Affiliations:** 1 Exercise Research Laboratory, Physical Education School, Federal University of Rio Grande do Sul, Porto Alegre, Brazil.; 2 Department of Health Sciences, Public University of Navarre, Spain.; 3 Faculty of Physical Education, University of Brasília.; 4 Sports Sciences, Brunel University of West London.; 5 Department of Nutrition, Federal University of Health Sciences of Porto Alegre, Brazil.; 6 Exercise Pathophysiology Research Laboratory, Clinical Hospital of Porto Alegre, RS, Brazil.

**Keywords:** surface EMG, fatigue, concurrent training, aerobic exercise, maximal strength

## Abstract

This study aimed to investigate the acute effects of two strength-training protocols on the neuromuscular and cardiorespiratory responses during endurance exercise. Thirteen young males (23.2 ± 1.6 years old) participated in this study. The hypertrophic strength-training protocol was composed of 6 sets of 8 squats at 75% of maximal dynamic strength. The plyometric strength-training protocol was composed of 6 sets of 8 jumps performed with the body weight as the workload. Endurance exercise was performed on a cycle ergometer at a power corresponding to the second ventilatory threshold until exhaustion. Before and after each protocol, a maximal voluntary contraction was performed, and the rate of force development and electromyographic parameters were assessed. After the hypertrophic strength-training and plyometric strength-training protocol, significant decreases were observed in the maximal voluntary contraction and rate of force development, whereas no changes were observed in the electromyographic parameters. Oxygen uptake and a heart rate during endurance exercise were not significantly different among the protocols. However, the time-to-exhaustion was significantly higher during endurance exercise alone than when performed after hypertrophic strength-training or plyometric strength-training (p <0.05). These results suggest that endurance performance may be impaired when preceded by strength-training, with no oxygen uptake or heart rate changes during the exercise.

## Introduction

Concurrent strength and endurance training is considered an optimal stimulus to promote both neuromuscular and cardiovascular gains (Cadore et al., 2012; Izquierdo et al., 2001; Mikkola et al., 2007; Silva et al., 2012). However, some studies have shown that concurrent training may result in lower strength gains compared with strength training alone, and this phenomenon is called the “interference effect” (Bell et al., 2000; Leveritt et al., 2003; McCarthy et al., 2002).

Regarding endurance adaptations, several studies have shown that there are no differences in the magnitude of cardiorespiratory adaptations when endurance training is performed alone or combined with strength training (Bell et al., 2000; McCarthy et al., 2002). However, Chtara et al. (2005) observed that performance of strength prior to endurance training resulted in lower enhancements in maximal endurance performed compared with the inverse order, which was justified as a consequence of fatigue resulting from strength training. Opposite results were observed by Cadore et al. (2012), who observed no influence of intra-session exercise order on maximal aerobic power in the elderly. Therefore, there is controversy regarding the effect of intra-session exercise order on endurance adaptations.

Only few studies, however, have investigated the acute effects of strength training on subsequent endurance performance, and these studies have focused on the effects of strength training on oxidative metabolism during endurance exercise (Alves et al., 2012; Kang et al., 2009), or on the effects of inducing muscle damage using eccentric protocols on endurance performance (Marcora and Bosio, 2007; Paschalis et al., 2005a, 2008; Twist and Eston, 2005). However, the acute effects of traditional methods of strength training, including training focusing on muscle hypertrophy or muscle power enhancements, on subsequent endurance exercise have been poorly investigated. In addition, in previous studies investigating the physiological effects of strength training on subsequent endurance exercise, the rest intervals between different parts of training (i.e., strength and endurance) lasted, at least, one hour (Alves et al., 2012; Kang et al., 2009). Therefore, it would be interesting to assess acute physiological effects of strength training on the subsequent endurance exercise when the last is performed immediately after the former. This investigation could bring relevant information about the physiological responses during endurance exercise when this modality is performed after strength exercises, as well as regarding the possible influence of strength training on the heart rate during endurance exercise, because the heart rate is often used as an intensity parameter during endurance exercise.

Therefore, the purpose of this study was to investigate the effects of two types of strength training sessions aimed at developing muscle hypertrophy and muscle power on subsequent neuromuscular and endurance performance. Our hypothesis was that both methods of strength training would impair endurance performance. In order to test our hypothesis, we chose to investigate the effects of two types of strength training on subsequent endurance performance: hypertrophic and plyometric strength training. The rationale for the use of these two training sessions was because first, strength training is often performed simultaneously with endurance training by practitioners looking for muscle hypertrophy and health promotion; and second, plyometric training is commonly performed by endurance athletes who aim to improve endurance performance (Paavolainen et al., 1999).

## Material and Methods

### Experimental Design

Participants in the present study attended the laboratory on several occasions. On the first visit, anthropometric measurements were assessed, and a maximal incremental test was performed on a cycle ergometer to determine maximal oxygen uptake. On the second visit, maximal strength was assessed using the one repetition maximum test. In the last visits, different exercise sessions were performed randomly, with one week of rest between sessions. In two of them, a strength training protocol (i.e., hypertrophic or plyometric) was performed before the endurance exercise protocol and in another, the endurance exercise protocol was performed alone. During each exercise session, physiological markers such as oxygen uptake and the heart rate, as well as the time to exhaustion (TTE) were assessed and compared. In addition, maximal isometric voluntary contractions (MIVC), the rate of force development (RFD) and EMG parameters related to neuromuscular fatigue such as EMG amplitude, median frequency, and the Dimitrov’s spectral fatigue index, were assessed before and after each strength training session. The ambient conditions were kept constant during all of the tests (temperature: 22–24° C). In addition, a food diary was completed two days before each training session, and the caloric intake on training days was controlled. These training sessions were performed at the same time in the morning (between 9 and 11 a.m.). All of the participants were experienced with strength and endurance exercises as well as with the maximal tests performed.

### Subjects

Thirteen healthy young and physically active men (age: 23.2 ± 1.6 years; body height: 176.4 ± 6.6 cm; body mass: 72.7 ± 7.1 kg; squat 1 RM = 112.2 ± 12.2 kg; VO_2max_ = 32.4 ± 4.7; n =13) volunteered for the study. The participants were used to performing three strength training sessions per week with the objective of promoting muscle hypertrophy, as well as to performing low intensity endurance training twice per week. The volunteers were carefully informed about the design of the study with information given regarding the possible risks and discomfort related to the procedures. After a verbal explanation of procedures, subjects signed an informed consent form. The study was approved by the Ethics Committee of the Federal University of Rio Grande do Sul and was performed in accordance with the declaration of Helsinki. Exclusion criteria included any history of neuromuscular, metabolic, hormonal and cardiovascular diseases. Subjects were not taking any medication with influence on neuromuscular metabolism.

### Peak oxygen uptake and ventilatory thresholds

To determinate the peak oxygen uptake (VO_2peak_), second ventilatory threshold (VT_2_) and workload at VT_2_ (W_VT2_) an incremental test on a cycle ergometer (Cybex®, USA) was performed. The test started at a 50 W load during two minutes and was progressively increased by 25 W each minute, until volitional exhaustion (McCarthy et al., 2002). The cycling cadence was maintained between 70–75 rpm. The expired gases were measured using a portable gas analyzer, model VO2000 (Medical Graphics®, Ann Arbor, EUA), and the data were recorded every 10 s. The VT_2_ was determined using the ventilation curve corresponding to the points of the exponential increase in ventilation in relation to the load and confirmed by ventilatory equivalent (VE/VCO_2_) (Wasserman, 1986). The load attained in the VT_2_ was accepted as W_LV2_. The peak VO_2_ obtained (ml·kg^−1^·min^−1^) - near exhaustion was considered the VO_2peak_. The heart rate (HR) was measured and registered every 10 s using a Polar monitor (Polar® model FS1, Shanghai, China). The test was considered valid if at least 2 of the 3 listed criteria were met: 1) the maximum heart rate predicted by age was reached (220-age); 2) the impossibility of continuing to pedal at a minimum velocity of 70 rpm; and 3) when RER greater than 1.1 was obtained.

### Maximal dynamic strength (1RM)

The 1RM was performed on the squat exercise using a multiforce machine (WORLD-Esculptor®, Porto Alegre, RS, Brasil) with 1 kg of resolution. On the test day, the subjects warmed up for five minutes on a cycle ergometer and performed specific movements for the exercise test. To avoid the influence of fatigue, each subject’s maximal load was determined with no more than five attempts with a four-minute recovery between attempts. The time for each contraction (concentric and eccentric) was 2 s, controlled by an electronic metronome (Quartz®, CA, USA). The test-retest reliability coefficient (ICC) was 0.99 for the 1RM squat test.

### Maximal isometric strength

To quantify maximal isometric strength, a load cell was fixed to multiforce machine (WORLD- Esculptor®, Porto Alegre, RS, Brasil) and connected to an A/D converter (Miotec®, Porto Alegre, Brazil). The subjects were positioned in the squat position at a knee angle of 90° (0° representing the full flexion). The subjects had two attempts at obtaining the maximum voluntary contraction (MIVC), each lasting 5 s, with a 1 min rest period between each attempt. Researchers provided verbal encouragement so that the subjects could exert possible maximum strength as fast as possible. This procedure was performed before and immediately after each strength training session (traditional and plyometric). MIVC was defined as the highest value of the force obtained during the 5 s contraction. The rate of force development (RFD) was determined from the slope of the force-time curve (Δforce/Δtime) of the isometric contractions over 10 ms time intervals. The test-retest reliability coefficient (ICC) was 0.94 for MVIC and 0.88 for RFD.

### Electromyography surface variables

Surface electromyography (EMG) data were recorded throughout the experimental protocol from the vastus lateralis and rectus femoris using a four-channel electromyograph (Miotool®, Porto Alegre, Brazil), with a sampling frequency of 2000 Hz per channel, connected to a personal computer (Dell Vostro 1000®, São Paulo, Brazil). Shaving and abrasion with alcohol were carried out on the muscular belly to ensure low impedance (above 2000 Ω) at skin-electrodes interface (Gonzalez-Izal et al., 2010). To ensure the same electrode position in subsequent tests, the right thigh of each subject was mapped for the position of the electrode moles and small angiomas by marking on transparent paper (Cadore et al., 2010). According to SENIAM EMG recommendations, in the vastus lateralis electrodes were placed at 2/3 on the line from the anterior spina iliaca superior to the lateral side of the patella; and, in the rectus femoris the electrodes were placed at 50% on the line from the anterior spina iliaca superior to the superior part of the patella. The ground electrode was fixed on the anterior crest of the tibia. The raw EMG signal was acquired during the MVIC. EMG signals were analyzed using Matlab software® (R2012, MathWorks Inc., USA). The following parameters were obtained from the sEMG signals for evaluation of neuromuscular fatigue: the root mean squared value (RMS), median frequency (MNF), and the Dimitrov spectral parameter (FInsm5) (Gonzalez-Izal et al., 2010). The RMS and median frequency were calculated in this study because these parameters are traditionally used to evaluate neuromuscular fatigue (Linnamo et al., 2000; Gonzalez-Izal et al., 2010; 2013). Additionally, the Dimitrov fatigue index was determined, because it appeared to be more sensitive to changes in the sEMG power spectrum than the mean or median frequencies (Dimitrov et al., 2006; Gonzalez-Izal et al., 2010). This parameter is obtained as the ratio between different moment orders (−1 and 5) and emphasizes the increases in the low and ultralow frequencies of the sEMG spectrum due to increased negative after-potentials as well as the decreases in high frequencies due to increments in duration of intracellular action potentials and decrements in action potential propagation velocity. The test-retest reliability coefficient (ICC values) of all EMG measurements was over 0.85.

### Caloric intake and nutritional report

The subjects were instructed not to change their eating habits and completed a nutritional record two days before each protocol. On test days, the subjects came to the laboratory in a fasting state and were supplemented with 1 g of maltodextrin per kilogram of body weight (g^−1^·kg^−1^) before each exercise session. The data collection procedures started one hour after the maltodextrin intake. In addition, a food diary was completed two days before each training session, and the caloric intake on training days was controlled. We assessed the nutritional status to ensure that the subjects started the different protocols with no differences on nutritional intake in the previous 48 hours to avoid possible influence of nutritional intake on exercise performance.

### Strength training protocols

On the days of exercise protocol, a warm-up of 10 repetitions of the squat exercise with the bar only was performed before both the hypertrophic and plyometric protocols. The hypertrophic strength training protocol (HT) was performed with a squat exercise using a multiforce machine (WORLD- Esculptor®, Porto Alegre, RS, Brasil). The participants performed 6 sets of 8 repetitions at 75% of their 1RM with a 2 min interval between sets. The performance time was 2 s for each phase of motion (i.e., concentric and eccentric).

The plyometric training protocol (PT) was composed of a countermovement jump exercise. The subjects were instructed to perform the eccentric and concentric phases at maximum speed possible, flexing the knee at an angle of approximately 90° before starting the concentric phase. Only body weight was used as the workload. The participants performed 6 sets of 8 repetitions of countermovement jumps with a 2 min rest interval between sets.

### Endurance exercise

The endurance exercise was performed on a cycle ergometer until volitional exhaustion. Initially, the subjects warmed up for 5 min at 25 W. Afterwards, the resistance was increased until the load corresponded to the second ventilatory threshold (W_VT2_) obtained during testing. During the protocol, a cadence between 70 and 75 rpm was maintained. Volitional exhaustion was considered when subjects were not able to maintain a cadence of 70 rpm or when they manifested the impossibility of continuing the task, so the protocol was suspended and the time of exhaustion recorded. In addition, the subjects were verbally encouraged during the protocol in order to motivate them to exert the maximal time of exercise as possible. During endurance exercise, VO_2_ and the HR were assessed and recorded in each 10 and 30 s of exercise, respectively, for further analysis. We calculated average VO_2_ and the average HR during the total time of exercise.

### Statistical analysis

The SPSS statistical software package was used to analyze all data. Normal distribution was checked with the Shapiro-Wilk test. Results are reported as mean ± SD. A comparison of the oxygen uptake, heart rate and time to exhaustion among different exercise protocols was performed using one-way ANOVA with repeated measures followed by Bonferroni post hoc analysis. The analysis of the neuromuscular parameters between strength training protocols (hypertrophy vs. power) in two moments (pre and post exercise) was performed using a two-way ANOVA (group × time). The retrospective statistical power provided by SPSS after analysis was over 0.90 in all variables. The level of significance was set at 0.05.

## Results

### Nutritional status

The comparison of the nutritional status among the training sessions showed no significant differences for total energy (p =0.621), carbohydrates (p =0.869), proteins (p =0.369) and fats (p =0.448) (Table 1).

### Cardiorespiratory and endurance performance variables

**Figure. 1:**
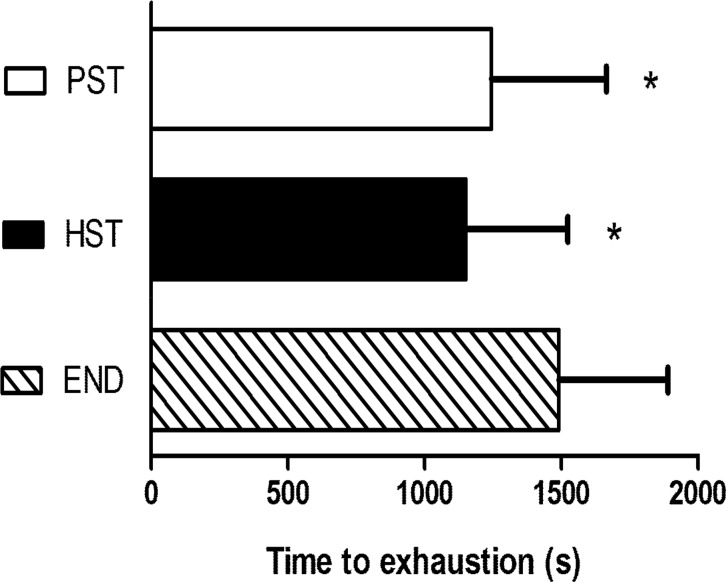
Time to exhaustion (s) (mean ± SD) during endurance exercise in the three protocols. PST, plyometric strength training prior to endurance training; HST, hypertrophy strength training prior to endurance training; END, only endurance training. *Significant difference from END (p =0.001).

There were no differences among the test days in VO_2_ and HR values before the start of endurance exercise. There were no significant differences among the protocols in mean VO_2_ values or mean HRs during the total time of exercise (Table 2). In addition, the TTE was significantly greater when endurance exercise was performed alone in comparison to both HST and PST protocols, with no differences between HST and PST (Figure 1 and Table 2).

### Neuromuscular variables

There were significant decreases in the MIVC after HST and PST, with no difference between the ST protocols (Table 3). In addition, significant decreases were also observed in the maximum rate of force development after HST and PST, with no difference between the ST protocols. Moreover, there were no significant changes in RMS values, median frequency or the Dimitrov’s spectral fatigue index in both the vastus lateralis and rectus femoris muscles after both ST protocols (Table 3).

## Discussion

The main finding of the present study was a decreased time to exhaustion on the cycle ergometer after both hypertrophic and plyometric training sessions, which may be related to the concomitant maximal strength and RFD decreases after the ST protocols. In addition, there were no changes in any of the neural activity parameters after the ST protocols or in the average oxygen uptake and heart rate during exercise. These findings expand the knowledge regarding the physiological responses and performance of endurance exercise during a concurrent training session and suggest that the HR may be used as an intensity parameter during endurance exercise after hypertrophic and plyometric training sessions. Moreover, hypertrophic ST or plyometric training sessions should be avoided immediately before endurance exercise if the aim of training is to maintain high-intensity endurance exercise for a prolonged time.

Regarding the physiological responses, our results corroborate the results found by Alves et al. (2012), who did not observe differences in VO2 or HR values during endurance exercise performed before and after a strength training session. In contrast, Kang et al. (2009) observed that VO2 was greater when endurance exercise was performed after strength training compared with the performance of endurance exercise alone, which was justified as a consequence of exercise post oxygen consumption (EPOC) resulting from ST contributing to the metabolic demand of the endurance exercise. In the present study, it was also possible that EPOC from the ST protocols was not sufficient to result in the average VO2 during endurance exercise. The discrepancies among the present results and those from previous studies may be a consequence of different methods used because in the study by Kang et al. (2009), the endurance exercise was performed only 5 min after strength training, instead of the 15 min interval used in the present study. Indeed, EPOC was greater in the first 5 min after the end of strength training and was thereafter markedly reduced in the following minutes of recovery (Almeida et al., 2001; Alves et al., 2012). In addition, in the study by Kang et al. (2009), the strength training session was composed of upper- and lower-body exercises instead of only one lower-body exercise as was used in the present study (i.e., squat or countermovement jump), which may have resulted in a lower metabolic demand during strength training and, consequently, lower EPOC. Moreover, in the study by Kang et al. (2009), aerobic intensity was 50% of VO2peak, while in the present study, the intensity corresponded to the second ventilatory threshold. Finally, although in the study by Kang et al. (2009), the ST session resulted in greater VO2 during endurance exercise in women, the same result was not observed in men, which is in accordance with the present results. Thus, along with the strength training protocols used, different populations may also explain different results.

Although no physiological differences were observed during different exercise protocols, there was a significant reduction in time to exhaustion when ST preceded endurance exercise (23% after HST and 17% after PST). Thus, at the same intensity, even with no VO2 changes, high-intensity endurance exercise was performed for a longer time (until exhaustion) when performed alone. It has been shown that after eccentric resistance exercise, there is a decrease in endurance performance as a result of muscle damage until 48 hours after the eccentric bout (Marcora and Bosio, 2007; Paschalis et al., 2005). Although the present study did not use a resistance exercise purely eccentric, the high workload performed in the HST protocol, and the characteristic of the PST protocol, which include high eccentric overload, may explain the impaired endurance performance when endurance exercise was performed after strength training.

However, to the best of the authors’ knowledge, this is the first study to compare the acute effects of performing different types of strength training on high-intensity endurance performance. Previously, Chtara et al. (2005) showed that strength training performed prior to high-intensity running (i.e., at VO2max velocity during 50% of pre-training time-to-exhaustion at this velocity) resulted in lower endurance performance than the inverse order. Interestingly, after training, the time to exhaustion in the group that performed strength training prior to endurance training was lower than in the inverse order (346 vs. 417 s) (Chtara et al., 2005). Although we did not investigate the chronic effects of performing strength training prior to endurance exercise in the present study, one could suggest that after a training period, the lower time to exhaustion during endurance exercise that was observed after the HST and PST protocols could result in lowered cardiorespiratory fitness gains. However, our results should be interpreted with caution because endurance exercise was performed at high intensity (i.e., at a workload that corresponded to the second ventilatory threshold), and the time of endurance exercise was until failure. Thus, if the time of exercise were lower (i.e., pre-determined), it is possible that no differences would be detected among the protocols because the HRs and VO2 values were similar among the protocols.

To identify possible neuromuscular mechanisms related to the impaired endurance performance after ST protocols, we assessed neuromuscular parameters such as MIVC, RFD, EMG amplitude, median frequency, and the Dimitrov’s spectral fatigue index. In the EMG parameters, no changes were observed in the RMS, median frequency and Dimitrov’s fatigue index values (Gonzalez-Izal et al., 2012). Regarding EMG amplitude, studies have often shown increases in these values (Izquierdo et al., 2009) under fatigue conditions, whereas decreases (Stephens and Taylor, 1972) and an absence of change (Linnamo et al., 2000; McCaulley et al., 2009) have also been observed. In the study by Linnamo et al. (2000) investigating neuromuscular responses to explosive (40% of 1 RM) and heavy (70% of 1 RM) resistance loads, no changes were observed in EMG amplitude values, whereas only slight increases were observed in the median frequency during the explosive type protocol. In addition, it has been shown that 5 sets of 10 RM (repetitions until concentric failure) lead to significant decreases in median frequency and increases in the Dimitrov’s fatigue spectral index (Gonzalez-Izal et al., 2010), which was not observed in the present study. A possible explanation of these different results could be that changes in median frequency and the Dimitrov’s fatigue index were observed during dynamic rather than isometric contractions in Linnamo et al.’s study (2000), in contrast to the assessments in the present study. In fact, recently it has been shown that changes in EMG parameters (amplitude, median frequency and Dimitrov’s index) are greater during dynamic rather than isometric contractions when the fatigue protocol is performed dynamically (Gonzalez-Izal et al., 2013).

In contrast, there were marked decreases in the MIVC (15% and 17% after HST and PST, respectively) and RFD (23% and 18% after HST and PST, respectively), which is in agreement with previous studies investigating fatigue after explosive and hypertrophic strength training protocols (González-Izal et al., 2010; Izquierdo et al., 2009; Linnamo et al., 2000; McCaulley et al., 2009). The MIVC and RFD are force outcomes strongly associated with endurance performance (Cadore et al., 2012; Paavolainen et al., 1999), which may help to explain the impaired endurance performance after ST protocols. The acute decreases in the MIVC and RFD could reflect type II fibre fatigue and, consequently, a lower contribution of this type of fibre during high-intensity endurance exercise performed in concurrent training protocols. Although we were not able to detect changes in the neural parameters assessed, other mechanisms related to the neuromuscular system, including changes in the neuromuscular junction properties, muscle conduction velocity, and reduced glycogen stores in type II fibres, may have induced the impairment in endurance performance after HST and PST. It should be noted that the subjects performed the exercise protocols at the same nutritional status, as shown by the dietary record, and with the same caloric intake before the exercise. Thus, the results do not appear to be influenced by nutritional factors.

In summary, hypertrophic and plyometric ST sessions performed prior to endurance training may similarly impair the time to exhaustion during high-intensity endurance exercise. In addition, despite the acute endurance performance impairment, ST protocols did not influence average VO2 values or HRs during exercise. From a practical standpoint, the HR may be used as an intensity parameter during endurance exercise even when it is performed after hypertrophic and plyometric training sessions. However, if the purpose of training is to perform high-intensity endurance training for as long as possible (i.e., second ventilatory threshold), then both hypertrophic ST and plyometric training should be avoided immediately before endurance exercise.

## Figures and Tables

**Table 1:** Values are mean ± SD of the alimentary register accomplished before protocols

	END	HST	PST
	
Total Energy (kcal)	2173.4 ± 587.1	2177.6 ± 647.4	2036.4 ± 471.8
Carbohydrates (g)	270.3 ± 104.1	269.0 ± 107.8	276.5 ± 84.2
Proteins (g)	102.3 ± 31.8	101.9 ± 41.5	86.36 ± 21.8
Lipids (g)	74.9 ± 20.7	77.1 ± 27.7	66.77 ± 18.0

No significant differences.

**Table 2:** Values are mean ± SD of cardiorespiratory responses and performance during endurance exercise

	END	HST	PST
	
TTE (s)	1491 ± 399	1152 ± 372^*^	1244 ± 421^*^
HR (beats·min^−1^)	164.8 ± 8.6	168.2 ± 5.1	168.1 ± 9.5
VO_2_ (ml·kg·min^−1^)	27.7 ± 3.05	29.8 ± 3.3	26.9 ± 4.2

TTE, time to exhaustion; HR, heart rate; VO_2_, oxygen uptake; END, endurance exercise alone; HST, hypertrophic strength training protocol + endurance exercise; PST, plyometric strength training protocol + endurance exercise.

* Significant difference from the endurance only protocol (p<0.05).

**Table 3:** Values are mean ± SD of neuromuscular responses before and after strength training protocols.

	HST		PST	

	Pre	Post	Pre	Post
	
MVIC (kg)	98.2 ± 8.9	83.8 ± 8.2^**^	111.15 ± 11.6	91.7 ± 11.5^**^
RFD (N·s^−1^)	548 ± 72.2	420.6 ± 46.5^*^	448.2 ± 58.5	369.0 ± 40.1^*^
RMS VL (μV)	190.2 ± 81.4	175.9 ± 51.0	204.1 ± 84.6	182 ± 73.1
RMS RF (μV)	154.7 ± 76.0	146.88 ± 57.28	161.4 ± 63.5	149.18 ± 70.1
Fmedian VL (Hz)	85.4 ± 6.9	85 ± 6	88.3 ± 17.5	86.7 ± 20.2
Fmedian RF (Hz)	99.6 ± 14.1	101.7 ± 19	97.1 ± 11.8	98.2 ± 15.5
FInsm5 VL (Hz^−6^)(10^−10^)	0.0027 ± 0.0019	0.0022 ± 0.001	0.0019 ± 0.0007	0.0025 ± 0.0011
FInsm5 RF (Hz^−6^)(10^−10^)	0.0028 ± 0.0021	0.0017 ± 0.0007	0.0023 ± 0.001	0.0023 ± 0.0012

MVIC, maximal voluntary contraction; RFD rate of force development; RMS root mean squared; Fmedian, median frequency; Flnsm5, Dimitrov’s spectral fatigue index, VL, vastus lateralis; RF, rectus femoris; HST, hypertrophic strength training protocol + endurance exercise; PST, plyometric strength training protocol + endurance exercise.

** Significantly different from pre ST protocol values (p< 0.01).

* Significantly different from pre ST protocol values (p <0.05).
